# Sensitivity to betrayal and new intimate relationship building in survivors of intimate partner violence

**DOI:** 10.1111/papt.70004

**Published:** 2025-08-08

**Authors:** Alice Melin, Paul M. Salkovskis

**Affiliations:** ^1^ The Oxford Institute of Clinical Psychology Training University of Oxford Oxford UK; ^2^ Department of Experimental Psychology University of Oxford Oxford UK; ^3^ Oxford Centre for Psychological Health, Oxford Health NHS Foundation Trust Warneford Hospital Oxford UK

**Keywords:** betrayal, intimate partner violence, relationships, self‐criticism, shame

## Abstract

**Objectives:**

There is evidence that prior experience of intimate partner violence (IPV) can lead to high levels of sensitivity to betrayal, shame and self‐criticism and interfere with initiation, development and maintenance of future intimate relationships. We measured these variables in women survivors of IPV, evaluating whether they are associated with the quality of current relationships.

**Design:**

A cross‐sectional, between‐groups design was used, comparing women survivors of IPV divided into those satisfied with current intimate relationships, those dissatisfied and IPV survivors not in such a relationship. Women without a history of IPV were included as a benchmark group.

**Method:**

Four groups: IPV single (*N* = 34), IPV dissatisfied (*N* = 25), IPV satisfied (*N* = 32) and those who had not experienced IPV (*N* = 42) were compared for betrayal sensitivity, followed by a secondary comparison of shame and self‐criticism. Online questionnaires were completed by participants recruited through social media and screened for IPV and relationship status.

**Results:**

All IPV groups had significantly higher scores for betrayal sensitivity than the non‐clinical group, with IPV satisfied having significantly lower scores than other IPV groups in two subscales: betrayal causing life change and lack of trust due to betrayal.

**Conclusions:**

Betrayal sensitivity is prominent in survivors of IPV, with evidence of a specific link between survivors' relationship satisfaction/status and their lack of trust and ideas of being permanently changed. Those appraisals may make it more challenging to build and maintain satisfactory relationships, or positive relationships may help survivors change their appraisals about betrayal, leading to a lack of trust and life‐altering changes.

## INTRODUCTION

### Intimate partner violence

Intimate partner violence (IPV) is a worldwide phenomenon, with 30% of women around the world having experienced IPV, and about 25% in Europe and 23% in rich countries (World Health Organization, 2013). It is defined by the World Health Organization (WHO) as a ‘behaviour within an intimate relationship that causes physical, sexual or psychological harm, including acts of physical aggression, sexual coercion, psychological abuse and controlling, coercive, repeated behaviours’ (WHO, 2010). This abuse may be perpetrated by men and by women towards romantic partners of the same or opposite sex.

IPV is listed within DSM‐5 under ‘Other conditions that may be a focus of clinical attention’ (American Psychiatric Association, 2013) as one of the relational problems that may characterise intimate adult partner relationships. As such, IPV is not a diagnosis or an intra‐psychic concept, which can make it difficult to find studies and theoretical models that investigate the clinical relevance of such experiences and has led to its conceptualisation more broadly under the umbrella of ‘trauma’ or ‘interpersonal trauma’. This in turn means that IPV research, like other trauma literature, has focused mainly on the intrapsychic negative sequelae of the experience by tracking associations with depression, posttraumatic stress disorder and anxiety (Samios et al., 2020). This could detract from a more inclusive understanding of a variety of psychological and relational responses (Elderton et al., 2015). An important consideration for many of those who have experienced IPV is the development (or not) of future intimate relationships.

Although there is little research on the experiences of IPV and new relationship building, it has consistently been suggested that domestic violence survivors experience significant relationship consequences, such as reduced likelihood of marrying and cohabitating (Burton et al., 2009; Cherlin et al., 2004; Macmillan, 2001; Manning et al., 2010; Vandervoort & Rokach, 2004). Research into the details of interpersonal difficulties following IPV and into rebuilding relational aspects of their lives in the medium to long term has largely focused on identifying risk factors that may predict and prevent revictimisation. This focus has in large part been determined by the coordinated global effort from the WHO and other charities to reduce IPV and more specifically violence against women, but one may question the utility of an ‘aetiological’ approach for psychologists providing individual one‐to‐one therapy to survivors rebuilding their lives. There are few longitudinal studies with survivors of domestic violence which means that little is known about how they establish new intimate relationships. However, a recent longitudinal study (Kelly et al., 2014) has found that most survivors in their study chose to remain single to stay safe rather than risk entering a potentially abusive relationship. It would appear that fears of revictimisation may not only be the main concern for the world health institutions but also for survivors, and the mechanisms by which it impacts survivors' ability to find and trust new partners safely (Kelly et al., 2014) are poorly understood.

### Relationship avoidance as self‐protection

The experience of IPV affects survivors' trust in future relationships as they may anticipate further betrayal experiences from intimate partners. Qualitative research suggests that survivors of IPV are indeed fearful of or expect violence when entering new relationships (Kelly et al., 2014; St. Vil et al., 2018). Similarly, in other betrayal traumas, survivors find it difficult to know whom and when to trust (Altmaier, 2016) and may be on high alert for possible new violations of trust. In order to protect themselves from revictimisation, IPV survivors have been shown to use a number of coping mechanisms that interfere with intimacy (Kelly et al., 2014; St. Vil et al., 2018). Experience of and sensitivity to betrayal may thus be a barrier to initiating, building and maintaining relationships. If this is so, it could be predicted that survivors who are highly sensitive to betrayal will be less likely to be in a fulfilling intimate partnership and vice versa.

### Identifying interpersonal trauma as betrayal trauma

The interpersonal nature of the trauma, where the stressor comes from the actions of a trusted person, has been highlighted as a factor that may account for the severity and chronicity of posttraumatic stress disorder (PTSD) (APA, 1994). Betrayal has been shown as a factor which could play a significant role in predicting psychopathology, including but not confined to PTSD (Freyd & Birrell, 2013). Betrayal may also explain aspects of aetiology (Kelley et al., 2012) and shed light on the mental health effects of exposure to IPV. Though physical wounds may heal, the emotional consequences of interpersonal trauma can persist and affect future relationships (Freyd et al., 2005; Platt et al., 2009). In her theory of Betrayal Trauma, Freyd suggests that it is the proximity of the relationship to the abuser that is crucial (Platt & Freyd, 2015). Thus, the conceptualisation of the way in which betrayal trauma has lasting effects may account for the mental health effects of interpersonal trauma and IPV.

The concept of betrayal has been widely studied, including in institutional betrayal (Brewin, 2003; Freyd, 1998; Shay, 2010) and a subtype of obsessive compulsive disorder (OCD), mental contamination (where the person feels contaminated internally by a current stimulus typically associated with the experience of betrayal; Pagdin et al., 2021; Rachman, 2010). Betrayal sensitivity is conceptualised here as how sensitive someone is to the possible experience of further betrayal and the negative impact of such betrayals, including but not confined to shame and self‐criticism.?

### Shame and self‐criticism in survivors of IPV

Shame and self‐criticism are common sequelae to interpersonal traumas, particularly betrayal traumas such as intimate partner violence and childhood abuse (Karakurt et al., 2014; Kubany et al., 1996). Betrayal trauma is associated with shame (Platt & Freyd, 2015), which in IPV is known to lead to beliefs about being to blame for ‘being a bad partner’ or ‘not having left sooner’ (Kubany & Ralston, 2008). Self‐criticism is also documented in survivors of IPV and has led to the development of interventions to promote self‐compassion in survivors, which has been shown to decrease following interpersonal traumas (Bistricky et al., 2017). Shame and self‐criticism predict greater prevalence of mental health difficulties including PTSD, depression and anxiety (Beck et al., 2015; O'Neill & Kerig, 2000), and may themselves negatively impact survivors' ability to form new relationships, linked to increased avoidance (Joseph et al., 1997). We therefore intend to identify the extent of shame and self‐criticism (and the relationship to betrayal sensitivity, relationship status and satisfaction).

There is thus evidence, which suggests that prior experience of IPV can lead to high levels of sensitivity to betrayal, shame and self‐criticism and therefore may interfere with initiation, development and maintenance of future intimate relationships. Understanding these interrelationships better could identify possible interventions and effective ways of supporting and empowering survivors to manage and (if they wish) to foster fulfilling partnerships. The current study aimed to examine levels of betrayal, shame and self‐criticism in three groups of women survivors of IPV those ‘in a relationship and satisfied’ group (described as ‘IPV satisfied’) with two other groups either ‘single’ (IPV single) or ‘not satisfied with their relationship’ (described as ‘IPV dissatisfied’) and an additional benchmark group of women who have not experienced IPV (non‐clinical group). We did not obtain data from women currently in relationship involving IPV.

In this study, we hypothesise that there will be a relationship between continuing sensitivity to betrayal and the development of new intimate relationships; that sensitivity to betrayal will be higher both in those with past experience of IPV who are currently single and in those dissatisfied in their current relationship relative to the other two groups. The secondary hypothesis is that self‐criticism and shame will also be higher in the IPV single and IPV dissatisfied group relative to the other two groups.

## METHOD

### Design

The study employed a between‐groups design using quantitative methodology to investigate sensitivity to betrayal, self‐criticism and shame across four groups with or without previous experience of IPV; IPV groups were defined in terms of their current relationship status, that is, IPV satisfied, IPV single, IPV dissatisfied and non‐clinical group.

### Recruitment

All participants were recruited online, focusing on online survivor and relationship groups through study advertisements in the form of digital flyers stating the anonymous and online nature of the survey, the aims of the study with links to the survey, and the department's online research page. This was posted to Twitter, Facebook and Instagram. Domestic violence organisations (i.e. Berkshire Women's Aid, and The Violence, Abuse and Mental Health Network VAMHN) and radio hosts (i.e. @litfriction) promoted the study through their online platforms. The researchers also posted the flyers to their own professional accounts and across several online community groups. A snowballing approach was also used, whereby participants were encouraged to share the study with others known to them.

### Selection criteria and participants

Individuals were eligible to take part if they were 18 or over, could read and write English and currently were or identified as a woman and were no longer in the IPV relationship.

Participants were not included if they self‐reported symptoms of psychosis and/or bipolar disorder, indicated that they were currently at risk from their new partner or the abusive relationship with their former partner had ended less than 1 year ago.

Participants were allocated to the non‐clinical group if they answered ‘NO’ to question ‘Have you ever been in an intimate partner violent relationship?’. Participants who answered ‘Yes’ to that question and ‘NO’ to the question: ‘Are you in a relationship?’ were allocated to IPV single group (*n* = 34). The remainder of the participants who answered yes to both questions were divided in two groups. It was decided a‐priori that IPV satisfied and IPV dissatisfied would be allocated to two groups based on an enhanced median split of their DAS‐7 scores. The median for this group was identified as 25, and those scoring more than two standard errors above and below were removed from the groups IPV used here: IPV satisfied *n* = 34 and IPV dissatisfied *n* = 25. Seven participants were not included for the between group analyses but are included in the regression analyses.

### Measures

Sociodemographic characteristics were also collected, including participants' age, gender, ethnicity, employment status and level of education. Additional questions focused on previous mental health difficulties, support and trauma history can be found in the Data S1. Validated study measures are detailed in Table 1.

**TABLE 1:** Overview of validated measures used in the study.

Domain	Measure
The Dyadic Adjustment Scale (DAS‐7), or Abbreviated Dyadic Adjustment Scale (ADAS; Hunsley et al., 2001)	The DAS‐7 is a 7‐item self‐report measure designed to assess the relationship quality of couples. This shortened version of the original from the original DAS‐32 (Spanier, 1976) includes items aimed at assessing relationship satisfaction and the degree to which the couple agrees on matters of importance to the relationship and is sensitive to change (Halford et al., 2012; Ireland et al., 2003; Zubrick et al., 2005). Scores below 21 indicate a relationship in distress (Hunsley et al., 2001). The DAS‐7 has established validity and good internal consistency with Cronbach's Alpha ranging from 0.76 (Sharpley & Rogers, 1984) to 0.84 in female participants (Hunsley et al., 1995), but no evidence relating to the test–retest reliability. Current study *α* = 712
The Perception of Betrayal Scale (POBS) (Pagdin et al., 2021)	A 27‐item questionnaire assesses the impact of betrayal on different aspects such as self‐perception, interpersonal relationships and behaviour. This measure was found to have high reliability (internal consistency) at Cronbach's alpha = .95. Preoccupation with past betrayal events subscale (*α* = .94), betrayal causing life change subscale (*α* = .89), lack of trust due to betrayal subscale (*α* = .88) and the betrayal leading to traumatic responses subscale (*α* = .81), all showed good reliability. Cronbach's alpha for the current study was *α* = .978. Preoccupation with past betrayal events subscale *α* = .963, betrayal causing life change subscale *α* = .961, lack of trust due to betrayal subscale *α* = .946 and betrayal leading to traumatic responses subscale *α* = .821
Forms of Self‐Criticism/Attacking and Self‐Reassuring Scale (FSCRS; Gilbert et al., 2004)	A 22‐item Likert scale developed to measure self‐criticism and the ability to self‐reassure. It is made up of three components including two forms of self‐criticalness: inadequate self and hated self; and one form of self‐reassurance. It has been found to have good validity and reliability (Baião et al., 2015). It has showed good internal consistency with *α* = .90 for inadequate self and .86 for hated self and reassured self respectively. In the current study all subscales showed good internal consistency: inadequate self *α* = .919, hated self *α* = .856, and reassure self *α* = .894
Other as Shamer Scale (OAS) (Goss et al., 1994)	The OAS is a valid and reliable measure of external shame (i.e. global judgements of how people think others view them). It is an 18 item, self‐report, 5 point Likert scale, with Cronbach's alpha: .92 (Goss et al., 1994). Current study *α* = .958
Experiences of Intimate Partner Violence	A 9‐item questionnaire created for the purpose of this study to identify/record the different types of violence and abuse experienced by survivors in historic intimate partnerships. This includes physical (e.g. ‘*I sustained physical injuries following an argument or fight with my former partner*’), psychological (e.g. ‘*My former partner used threats and intimidation*’) and sexual aggression, as well as digital abuse (e.g. ‘*My former partner posted unwanted photos and videos of a sexual nature on the internet*’).

### Procedure

Data were collected through an anonymous online Qualtrics survey software. Participants accessed the survey via weblinks included on study advertisements and were presented with a Participant Information Sheet (PIS) detailing risks and benefits of taking part in the study. Upon confirming the consent form and inclusion criteria, participants progressed to demographic questions. The following questions about IPV experience and relationship helped to pre‐allocate groups. Exclusion criteria questions (e.g. risk from current partner) came up to ensure participants met the criteria and avoid the collection of irrelevant data. All participants in a relationship were asked to complete a relationship measure; this measure's total score helped to determine allocation to IPV satisfied or dissatisfied. Single women were not asked to answer questions about relationship satisfaction. On the last page, a debrief message was provided, including a grounding exercise, contact details for accessing mental health and domestic violence support. Two women with lived experience of intimate partner violence were consulted regarding the design, procedure, materials and impact of the study to acknowledge the value of service users' contributions to research development (National Institute for Health Research, 2018).

Data collection took place from January to May 2022 with ethical approval from the University of Oxford Central University Research Ethics Committee (R78380/RE001).

### Power calculation

Power analysis was conducted drawing from a study (Howkins et al., 2020) investigating sensitivity to betrayal and mental contamination using a similar methodology and analysis to the one proposed by this study. G*Power software indicated that for an ANOVA with total betrayal sensitivity as the primary dependent variable and an estimated effect size of cohen *f* = 0.3; alpha = 0.05 and power of 0.8, the minimum required sample size is estimated to be a total of 128 participants. Consequently, this study aimed to recruit at least 128 participants (32 per group) from the community. A total of 140 participants were eventually recruited.

### Data analytic strategy

Groups were compared based on chi‐square tests. For the purpose of statistical analysis, categories with smaller cell sizes were concatenated for age (collapsed into <36 and 36>), ethnicity (collapsed into ‘White’ and ‘Non‐White’), employment status (‘In work or study’ or ‘Not working’) and highest level of education (Academic Higher Qualification and Non‐academic).

Continuous data for the dependent variables were found to be normally distributed based on Kolmogorov–Smirnov tests. In mixed‐model ANOVAs, where the Mauchly test of sphericity was significant, the Greenhouse–Geisser correction was applied and is indicated in the text below. Where significant interactions were noted in mixed‐model analyses, these were followed up by simple main effects and multiple comparisons using Tukey LSD except where Levene's test for equality of variance was significant, in which case Dunnett's T3 non‐parametric comparisons were used.

To test the first hypothesis, that betrayal sensitivity will be higher in the IPV single and dissatisfied group, we conducted a mixed‐model analysis of variance (ANOVA), with betrayal sensitivity (POBS) subscale as the within‐subjects variable and group as the grouping variable.

To test the secondary hypothesis that self‐criticism and shame will be higher in the IPV single and IPV dissatisfied groups relative to the IPV satisfied group, we first conducted a mixed model ANOVA comparing self‐criticism subscales across the four groups and shame using a separate one‐way ANOVA between groups.

As an additional exploratory analysis, we conducted a hierarchical multiple regression with these three measures as IV and the participants DAS‐7 score as the DV.

## RESULTS

Demographic characteristics of each group are summarised in detail in the Data S1. The total number of participants enrolling was 140: 34 in the IPV single group, 25 in the IPV dissatisfied group, 32 in the IPV satisfied group and 42 in the non‐clinical group.

Chi‐square analyses indicated no significant difference between the four groups in ethnicity (*p* = .937) and employment status (*p* = .330). Significant associations were, however, found between groups in age (χ^2^ (1) = 20.703, *p* < .001) and education status (χ^2^ (1) = 17.129, *p* < .001). Using partitioned chi‐square analyses, it was found that IPV dissatisfied and single were significantly more likely than IPV satisfied to be 36 or above (χ^2^ (1) = 9.687, *p* = .002, χ^2^ (1) = 4.331, *p* = .037, respectively). No other significant differences were found for age and education for any of the partitioned analyses.

Significant associations were found between groups in self‐reported depression (χ^2^ (1) = 23.597, *p* < .001), anxiety (χ^2^ (1) = 8.559, *p* < .036) and PTSD (χ^2^ (1) = 25.551, *p* < .001). Using partitioned chi‐square analyses, it was found that the IPV single group was significantly less likely than IPV dissatisfied to report having experienced depression (χ^2^ (1) = 5.001, *p* = .025). No other significant differences were found for self‐identified depression, anxiety and PTSD on partitioned analyses.

Abusive experiences were each recorded with a score of 1 and added together to produce a total of 9 (Table 2). A one‐way ANOVA on IPV experiences between IPV groups found no significant effect of group *F*(2, 90) = 0.610, *p* < .545.

**TABLE 2:** IPV total mean scores by group.

	IPV single group *n* = 34 mean (SD)	IPV dissatisfied *n* = 25 mean (SD)	IPV satisfied group *n* = 32 mean (SD)
IPV experiences total	5.03 (2.07)	5.36 (2.06)	4.75 (2.08)

### Primary outcome variables

**FIGURE 1:**
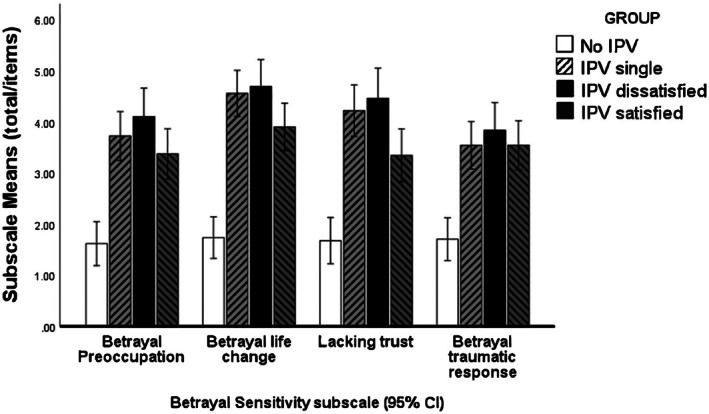
Betrayal sensitivity subscale scores shown by criterion group.

A mixed‐model ANOVA on betrayal sensitivity with subscales as the within‐subject variable and IPV group as grouping variable (Table 3) found a significant main effect of subscale type (*F*(2.310, 297.992) = 12.268, *Greenhouse–Geisser p* < .001). The main effect of group was also significant *F*(3, 129) = 35.297, *p* < .001. These effects were modified by a significant group by subscale interaction, (*F*(6.930, 297.992) = 2.653, *Greenhouse–Geisser p* < .001); this is illustrated in Figure 1.

**TABLE 3:** POBS subscales mean scores by group.

	IPV single group *n* = 34 mean (SD)	IPV dissatisfied *n* = 25 mean (SD)	IPV satisfied *n* = 32 mean (SD)	Non‐clinical group *n* = 42 mean (SD)
Preoccupation with betrayal events	37.29 (14.13)^a^	41.08 (11.05)^a^	33.78 (13.16)^a^	16.19 (16.18)^b^
Betrayal causing life change	31.94 (7.55)^a,c^	32.88 (5.85)^a^	22.34 (8.10)^c^	12.17 (12.49)^b^
Lack of trust due to betrayal	25.35 (7.79)^a,c^	26.80 (6.37)^a^	20.09 (10.29)^c^	10.07 (9.87)^b^
Betrayal leading to traumatic responses	14.18 (5.29)^a^	15.36 (4.79)^a^	14.19 (4.64506)^a^	6.83 (6.47)^b^

*Note*: Means which share subscripts are not significantly different (*p* > .05).

As the interaction was significant, simple main effects analyses for the POBS subscales were conducted by group. There was a significant main effect of group for all four subscales: preoccupation with betrayal (*F*(3, 132) = 22.341, *p* < .001), betrayal causing life change (*F*(3, 132) = 39.689, *p* < .001), lack of trust (*F*(3, 132) = 26.172, *p* < .001) and betrayal leading to traumatic responses (*F*(3, 132) = 19,107, *p* < .001).

Post‐hoc multiple comparisons (LSD) show that the IPV groups were significantly higher across all the subscales of the sensitivity to betrayal measures relative to controls. Post‐hoc multiple comparisons (Dunnett T3) also showed that the IPV satisfied group had significantly lower mean scores than IPV dissatisfied on two subscales (betrayal life change, *p* < .024; lack of trust, *p* < .023), but not on the subscales preoccupation with betrayal events and betrayal leading to traumatic responses. There were no other significant differences between the groups across the other POBS subscales.

### Secondary outcome variables

A mixed‐model ANOVA was used to conduct an analysis on FSCRS (self‐criticism) with the subscales (inadequate self, reassure self, hated self) as within‐subject variables and IPV group as the grouping variable (Table 4).

**TABLE 4:** FSCRS subscales mean scores by group.

	IPV single group *n* = 34 mean (SD)	IPV dissatisfied *n* = 25 mean (SD)	IPV satisfied group *n* = 32 mean (SD)	Non‐clinical group *n* = 42 mean (SD)
FSCRS inadequate self	20.03 (9.77)^a,b,c^	23.32 (9.31)^a^	18.25 (8.38)^b,d^	17.26 (9.31)^b,c,d^
FSCRS reassure self	13.79 (6.05)^a^	13.52 (6.80)^a^	17.50 (6.98)	21.04 (6.55)
FSCRS hated self	6.06 (5.36)^a,b^	7.88 (5.63)^a^	4.37 (4.57)^b,c^	3.28 (4.45)^c^

*Note*: Means which share subscripts are not significantly different (*p* > .05).

The ANOVA indicated a significant main effect of subscale *F*(1.226, 158.108) = 121.646, *Greenhouse–Geisser p* < .001. The main effect of group was not significant, *F*(3, 129) = 1.34, *p* = .265.

**FIGURE 2:**
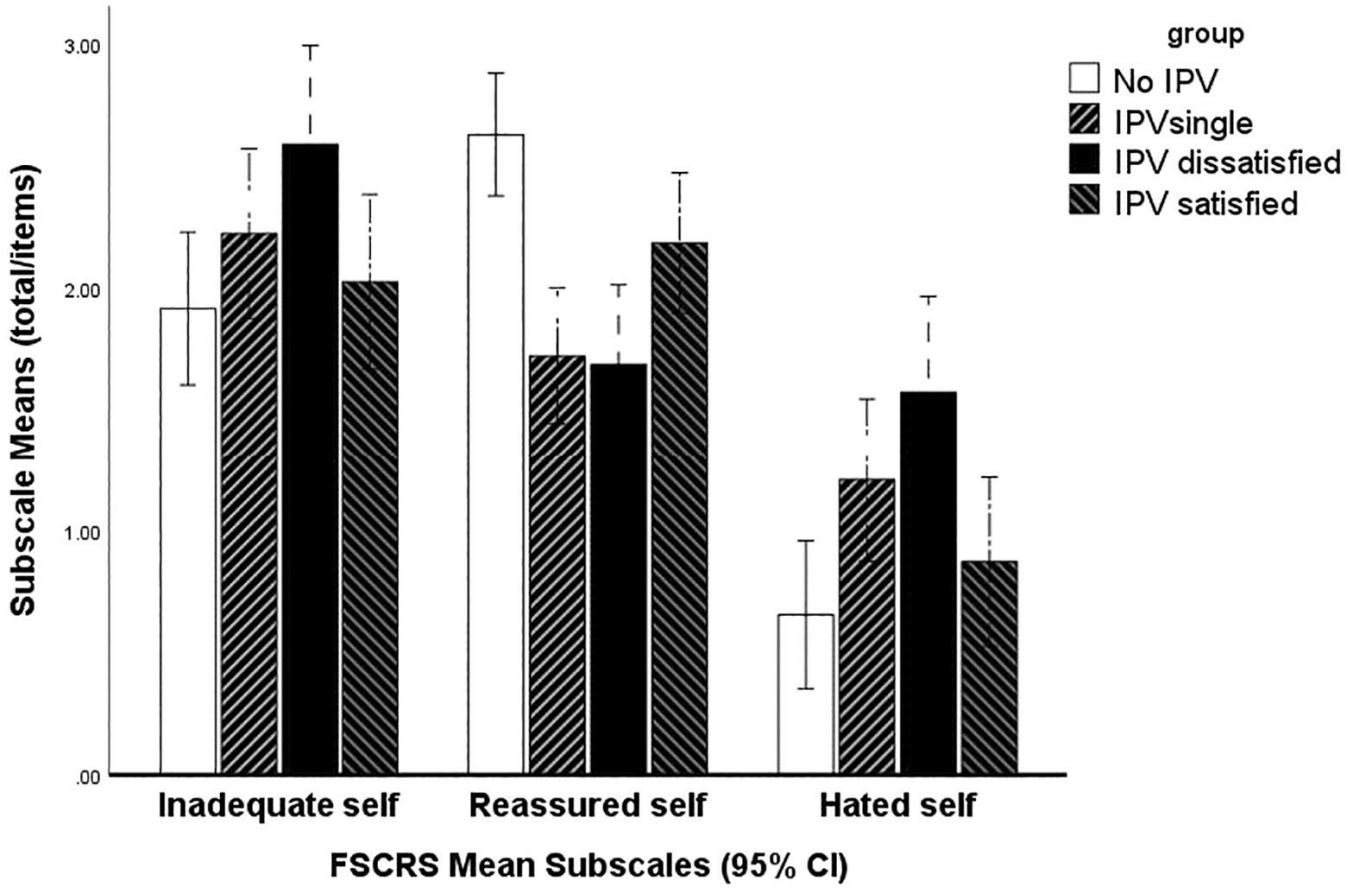
Self criticism (FSCRS) subscale scores across groups.

The main effect of subscale was modified by a significant group by subscale type interaction (*F*(3.677, 158.108) = 6.224, *Greenhouse–Geisser p* < .001); this is illustrated in Figure 2.

Simple main effect analyses for the FSCRS subscales were conducted to decompose it; two subscales indicated a main effect of group. Reassure self (*F*(3, 132) = 10.370, *p* < .001) and hated self (*F*(3, 132) = 5.147, *p* < .001) but not inadequate self (*p* = .064).

Post‐hoc multiple comparisons (LSD) showed that IPV dissatisfied groups were significantly higher across the inadequate self than IPV satisfied (*p* < .05) and controls (*p* < .05).

The IPV dissatisfied (*p* < .001) and single (*p* < .001) groups were significantly lower across the subscale reassure self than controls (*p* < .001) and than IPV satisfied (*p* < .05). IPV satisfied was also significantly lower than controls (*p* < .05).

The IPV dissatisfied (*p* < .001) and single (*p* < .001) groups were significantly higher than controls (hated self *p* < .05) and IPV dissatisfied was significantly higher than satisfied (hated self *p* < .05). There were no other significant differences.

#### Shame

A one‐way ANOVA was used to conduct an analysis on external shame between IPV groups (see Table 5). There was a significant effect of group *F*(3, 132) = 8.880, *p* < .001.

**TABLE 5:** OAS mean scores by group.

	IPV single group *n* = 34 mean (SD)	IPV dissatisfied *n* = 25 mean (SD)	IPV satisfied group *n* = 32 mean (SD)	Non‐clinical group *n* = 42 mean (SD)
Other as Shamer Scale	34.68 (15.36)^a,b^	34.76 (12.43)^a,c^	32.03 (16.48)^b,c^	20.16 (12.66)

*Note*: Shared superscripts indicate groups not significantly different, *p* > 0.05.

Post‐hoc comparisons using LSD test showed OAS total was significantly lower in all IPV groups relative to the non‐clinical groups, (*p* < .05) which did not differ from each other.

#### Regression

A multiple linear hierarchical regression analysis for the full sample with relationship satisfaction as the DV was run, entering shame and self‐criticism in the first step (*F*(2, 62) = 12.593, *p* < .001) *R* ^2^ = .266. Sensitivity to betrayal was entered in the second step, giving (*F*(3, 61) = 13.454, *p* < .001) *R* ^2^ = .369, indicating that the sensitivity to betrayal scale accounted for an additional 10.9% of the variance (Table 6).

**TABLE 6:** Multiple regression results for FSCRS and FSCRS predicting DAS‐7 scores, and with added POBS predicting DAS‐7 scores.^a^

Variable	Beta	*t* value	*p* value
Model 1 FSCRS	.30	0.241	.811
Model 1 OAS	−.553	−4.407	<.001
Model 2 FSCRS	.01	0.006	.996
Model 2 OAS	−.210	−1.352	.181
Model 2 POBS	−.466	−3.329	<.001

^a^ Includes the participants who were removed for the IPV satisfied and IPV dissatisfied group division.

## DISCUSSION

This study sought to evaluate the extent to which sensitivity to betrayal differed between IPV survivors who have engaged in new and positive intimate relationships and those who have not, and how this related to self‐criticism and shame. Results indicated that high betrayal sensitivity was present in all IPV survivors relative to controls, but that there was also some evidence of specificity, with IPV‐dissatisfied group scoring higher on the subscales ‘permanent change’ and ‘lack of trust’ compared to both the IPV‐satisfied and non‐IPV groups, but not compared to the IPV‐single group. It was anticipated that survivors of IPV who are in a relationship and satisfied would show less sensitivity to betrayal than those dissatisfied in a relationship or single. Although this was so for the ‘betrayal life change’ and ‘lack of trust’ subscales, the IPV satisfied group was not significantly different from IPV dissatisfied or IPV single groups for the subscales ‘preoccupation with betrayal events’ and ‘betrayal leading to traumatic responses’. With regards to the self‐criticism and shame hypothesis, only partial support was found. The IPV‐dissatisfied group had significantly and similarly higher scores on all aspects of the scale (‘inadequate self’, ‘hated‐self’ and ‘reassured‐self’) compared to IPV‐satisfied and controls, meaning they were more self‐critical and less able to remind themselves of positive things about themselves. The IPV single group were also less able to reassure themselves than IPV‐satisfied and controls and were only significantly more self‐critical in hated‐self compared to controls. IPV‐satisfied were only significantly different from controls on their ability to reassure themselves, meaning high self‐criticism was specific to IPV‐dissatisfied and IPV single. For shame, all three IPV groups scored higher than the non‐IPV group, but there were no significant differences within the three IPV groups.

These findings are consistent with research suggesting that betrayal trauma alters schemas about trust (Bistricky et al., 2017) and the way in which it impacts knowing and choosing who we do trust (Altmaier, 2016). The present study cannot determine whether aspects of low betrayal sensitivity in the IPV satisfied group has led to that better relationship, or whether the experience of being in a satisfactory relationship has helped survivors in the IPV satisfied group increase their trust and reduced betrayal sensitivity. Appraisals of trauma sequelae such as being permanently changed are common in survivors with PTSD and play a key role in predicting chronicity (Ehlers & Clark, 2000). Relationship satisfied survivors' positive relationships may have altered these appraisals, being in a satisfactory relationship may be taken as evidence by the survivor that the impact of the trauma has not led to complete permanent life change. Alternatively, one could hypothesise that the belief that others are trustworthy is essential to the formation of a successful intimate partnership, and that thus only the individuals with more positive beliefs about trusting others have been able to build such satisfactory relationships (Burton et al., 2009; St. Vil et al., 2018). Furthermore, believing that oneself and one's life is permanently changed could be an unhelpful belief to hold, acting as a barrier to building and maintaining good relationships. In fact, research suggests that a pessimistic view about people and the world can be a consequence of betrayal trauma (Freyd et al., 2005), however holding a more optimistic view may be essential to those fulfilling relationships.

It is possible (and would be unsurprising if true) that the dissatisfaction of survivors in their new relationships could be related to their increased difficulties with trust. It could also be evidence of misplaced trust, with some of those survivors having trusted their partners and entered into these relationships too quickly (Burton et al., 2009), and this may have negatively impacted their choice of partner or the relationship itself, leading to current dissatisfaction. Control strategies deployed in response to beliefs about being permanently changed and others not ever being trustworthy may also lead to counter‐productive behaviours, such as compartmentalised trust and enforced emotional distance between themselves and their partners to protect themselves from vulnerability to further IPV and future betrayals (Cherlin et al., 2004). Such strategies may be protective but may also generate relationship difficulties. Most likely this group requires finer‐grained consideration accounting for different reasons for remaining single.

Participants in the single and dissatisfied groups demonstrated similar levels of sensitivity to betrayal as those of depressed patients and low MC in a recent study (Howkins et al., 2020). We know that depression is more likely in IPV survivors (Devries et al., 2013), potentially up to three times more (Golding, 1999). It could be that IPV survivors suffering from depression are more likely to negatively view their relationship as well as have an increased sense of betrayal, or simply that betrayal sensitivity is a generalised factor in the experience of mental health difficulties. Further research is needed to better understand this interplay.

### Limitations

The sample included a high proportion of participants with postgraduate qualifications, which perhaps exemplifies how the homophily principle can lead to a biased sample when recruiting through social networks (McPherson et al., 2001). However, it has also yielded data around a group of survivors who are less often seen in services and suggests that IPV is experienced across socio‐economic status, challenging preconceptions that IPV is prevalent in lower social classes. This being a self‐selecting group, it may be that those with academic or research experience may have been more interested in donating their time to research or found the study more accessible. Due to the small sample size and its lack of diversity across ethnicity (predominantly white) and exclusion of those with bipolar and psychosis diagnoses, one may argue that it is less reliable and generalisable to wider populations (Denscombe, 2010). Data were all derived from self‐report questionnaires; future research should consider developing structured interviews to measure the IPV variables. Determination of IPV status was through self‐identification; there is a clear need for a brief measure, which can more precisely identify IPV without traumatising those surveyed.

### Clinical implications

The findings add to evidence that survivors are fearful of future betrayals and therefore may need support rebuilding their confidence in knowing who and when to trust with a view to building new intimate partnerships. Conceptualising the impact of complex trauma as sensitivity to betrayal, more specifically difficulties with trust, may help understand how we establish alliances with traumatised service users and how therapeutic ruptures can be avoided and repaired. Interventions should consider these appraisals around trust being and permanently changed, and seek to measure them throughout treatment alongside the quality of their relationships.

### Future research

More research is needed into how sensitivity to betrayal manifests itself in survivors of different types of interpersonal traumas and experiences and affects their relationships. A longitudinal study with IPV in all groups would elucidate the nature of the relationship between these beliefs and survivors' relationship status and satisfaction. Mediational studies would be good to evaluate the role of the relationship on sensitivity to betrayal and to explore associated appraisals. Evaluation of a focused psychological intervention, such as schema‐focused therapy targeting betrayal beliefs, could help survivors develop satisfactory relationships after their traumatic experience of IPV, and in doing so provide a new avenue of supporting survivors in their long‐term recovery. More investigation into the role of shame and self‐criticism could inform therapeutic approaches and improve understanding of long‐term recovery in IPV.

## CONCLUSION

This research suggests that betrayal sensitivity is a useful construct to conceptualise some of the barriers IPV survivors face when building new intimate relationships. Further research is still needed to better understand the underlying mechanisms with a view to developing interventions that can reduce barriers to positive relationships and support survivors with long‐term recovery.

## AUTHOR CONTRIBUTIONS

**Alice Melin:** Conceptualization; investigation; writing – original draft; methodology; validation; writing – review and editing; formal analysis; project administration. **Paul M. Salkovskis:** Conceptualization; investigation; methodology; validation; writing – review and editing; formal analysis; project administration; data curation; supervision.

## FUNDING INFORMATION

No funding was received for this work.

## CONFLICT OF INTEREST STATEMENT

None known by either author.

## Supporting information


Data S1.

## Data Availability

The data that support the findings of this study are available on request from the corresponding author. The data are not publicly available due to privacy or ethical restrictions.
