# Supplementary material for: Sensitivity to betrayal and new intimate relationship building in survivors of intimate partner violence
Source: Psychol Psychother. 2025 Aug 8;98(4):1050–63. doi: 10.1111/papt.70004 (PMC12617496; doi:10.1111/papt.70004)
Supplement: Supplementary file 1 — Data S1. [file PAPT-98-1050-s001.docx]

**Supplementary document: descriptive characteristics**

Table 2. *Demographic characteristics of groups*

|  | IPV single group *n*= 34  N (%) | IPV dissatisfied *n*= 25  N (%) | IPV satisfied *n*=32  N (%) | Non-clinical Group *n*= 42  N (%) | IPV  *n*= 7  N (%) |
| --- | --- | --- | --- | --- | --- |
| **Age**  18 - 25 years  26 - 35 years  36 - 55 years  56 years +  Prefer not to say | 1 (2.9%)  8 (23.5%)  20 (58.8%)  5 (14.7%) | 1 (4.0%)  2 (8.0%)  18 (72.0%)  4 (16.0%) | 5 (15.6%)  11 (34.4%)  14 (43.8%)  1 (3.1%)  1 (3.1%) | 4 (9.5%)  22 (52.4%)  14 (33.3%)  2 (4.8%) | 1 (2.9%)  8 (23.5%)  20 (58.8%)  5 (14.7%) |
| **Age**  18-35  35+  Prefer not to say | 9 (26.5%)  25 (73.5%) | 3 (12%)  22 (88%) | 16 (50%)  15 (48.9%)  1 (0.03%) | 26 (61.9%)  15 (38.1%) | 9 (26.5%)  25 (73.5%) |
| **Ethnicity**  White  Non - white | 30 (88.2%)  4 (11.8%) | 22 (88%)  3 (12%) | 29 (90.6%)  3 (9.4%) | 36 (85.7%)  6 (14.3%) | 30 (88.2%)  4 (11.8%) |
| **Employment Status**  In work or study  Not working | 29 (85.3%)  5 (14.7%) | 19 (76.0%)  6 (24.0%) | 29 (90.6%)  3 (9.4%) | 38 (90.5%)  4 (9.5%) | 29 (85.3%)  5 (14.7%) |
| **Highest level of education**  Academic Higher Qualification  Non-academic | 22 (64.7%)  12 (35.3%) | 13 (52.0%)  11 (44.0%) | 25 (78.1%)  7 (21.9%) | 40 (95.2%)  2 (4.8%) | 22 (64.7%)  12 (35.3%) |
| **Depression**  Yes  No | 16 (47.1%)  18 (52.9%) | 19 (76%)  6 (24%) | 19 (59.4%)  13 (40.6%) | 8 (19%)  34 (81%) | 16 (47.1%)  18 (52.9%) |
| **Anxiety**  **Yes**  **No** | 19 (55.9%)  15 (44.1%) | 14 (56%)  11 (44%) | 21 (65.6%)  11 (34.4%) | 14 (33.3%)  28 (66.7%) | 19 (55.9%)  15 (44.1%) |
| **PTSD**  **Yes**  **No** | 15 (44.1%)  19 (55.9%) | 15 (60%)  10 (40%) | 12 (37.5%)  20 (62.5%) | 2 (4.8%)  40 (95.2%) | 15 (44.1%)  19 (55.9%) |
| **Relationship Status**  **In a relationship**  **Single** | 34 | 25 | 32 | 40  2 | 7 |

**†** does not include the participants who were removed for the IPV Satisfied and Dissatisfied group

Additional questions focused on previous mental health difficulties, support and trauma history, followed by the Dyadic Adjustment Scale (DAS-7), Experiences of IPV questions, and Perception of Betrayal Scale (POBS):

**Demographics characteristics**

**What is your age?**

- 18 - 25 years (1)
- 26 - 35 years (2)
- 36 - 55 years (3)
- 56 years (4)
- + Prefer not to say (5)

**What is your gender?**

- Male (1)
- Female (2)
- Other (3)
- Prefer not to say (4)

**What is your highest level of education?**

- GCSE level or equivalent (1)
- A-Level or equivalent (2)
- Undergraduate Degree (3)
- Higher Degree (Masters, PhD or equivalent) (4)
- Professional qualifications (5)
- Apprenticeship (6)
- Other vocational/work related qualification (7)
- No formal education (8)

**What is your employment status?**

- Employed full time paid (1)
- Employed part time paid (2)
- Self-employed or freelance (3)
- Other paid work (4)
- Unemployed and currently looking for work (5)
- Unemployed and not currently looking for work (6)
- Homemaker (7)
- Student (8)
- Volunteer (9)
- Retired (10)
- Unable to work (11)

**Have you experienced mental health difficulties currently or in the past?**

- Yes (1)
- No (2)

**Please tick any diagnoses given past or present below:**

- Depression (1)
- Anxiety (Generalised anxiety disorder/Panic disorder/Obsessive-compulsive disorder/Phobias etc… (2)
- Post-traumatic stress disorder/Complex PTSD (3)
- Personality disorder (4)
- Psychosis and/or schizophrenia, and/or bipolar disorder  (5)
- Other (6)
- Not Applicable  (7)

**Have you received mental health support currently or in the past?**

- Yes, I have received mental health support such as: Talking therapy (e.g. counselling, therapy, psychological etc…) Medication Both talking therapy and medication (1)
- No, I have never received mental health support (2)

**Have you experienced a traumatic event?
(Please tick all that are appropriate and happened before the recent abusive relationship referred to in this research)**

- Previous intimate partner violence (1)
- Sexual or Physical violence (2)
- Single event trauma (natural disaster, transport accident) (3)
- Childhood trauma (physical or sexual abuse, neglect, etc…) (4)
- Any other event where your life was in danger (5)

**How would you describe your ethnicity?**

- White British (1)
- Black British (2)
- Asian British (3)
- Other British (4)
- White Other (5)
- Black Other (6)
- Asian Other (7)
- Other (8)
- Prefer not to say (9)
- Latin American (11)
